# Literary Notes

**Published:** 1907-04

**Authors:** 


### LITERARY NOTES.

Folia Urologica is the title of a new international archives relating to genitourinary diseases. With Professor James Israel of Berlin as editor-in-chief, Professor A. Kollmann of Leipzig, Dr. G. Kulisch of Halle and Dr. W. Tamms of Leipzig as associate editors and the other principal urologists of Europe as collaborators, these new international archives are announced by the house of W. Klinkhardt, Leipzig. Exhaustive original articles with colored plates and illustrations will be the principal feature of *Folia Urologica.* Contributions will be published in the four languages that are officially used in Congresses and each paper will be summarized in the three other languages. The new publication will contain a department entitled "Events in Urology" in which the regular collaborators will periodically report on the advances in this specialty, after having tested them critically in their respective services and laboratories. Finally, *Folia Urologica* is to serve as a means of collecting the Annual Reports on urological work in hospitals, clinics, etc., throughout the world. With a view to publishing contributions as quickly as possible, the issues of *Folia Urologica* will appear as often as required. Contributions from North, Central and South American authors may be sent to either of the American editorial representatives, William N. Wishard, Newton-Claypool Building, Indianapolis, Ind., or Ferd. C. Valentine, 171 West 71st Street, New York.

---

THE JOURNAL OF INEBRIETY, after thirty years of continuous study of the disease of inebriety and drug taking, begins its new decade by entering upon the comparatively new field of physiological and psychological therapeutics, for the treatment of these neuroses. Arrangements have been completed by which *The Archives of Physiological Therapy* has been consolidated and will hereafter be published as a part of *The Journal of Inebriety.* This very able monthly has been developing parallel lines of study with *The Journal of Inebriety.* In the opinion of its managers its scientific value would be greatly enlarged by concentrating its work along some special lines. The disease of inebriety and its allied neuroses is a field of most practical interest, hence *The Journal of Inebriety* is selected as a medium for continuing the work of *The Archives of Physiological Therapy.*

Henceforth, in addition to the various phases of this subject which *The Journal* has presented, the therapeutic effects of hot air, radiant light baths, electricity, massage, psycho-therapeutic measures and other physiological means will occupy a prominent space. This effort to clear away the confusion and broaden the studies of therapeutic means for cure, will make *The Journal of Inebriety* one of the most practical and valuable visitors to every hospital and institution, as well as to all specialists who treat brain and nerve neurotics. We shall aim to present and formulate the latest studies and facts along these frontier lines, and in this way lift the whole field of therapeutics out of its present empiric stage into one of rational therapeutics.

